# Mesiobuccal2 (MB2) canal evaluation for maxillary first molar in Indian sub-population

**DOI:** 10.6026/973206300221820

**Published:** 2026-03-31

**Authors:** Rishu Singh, Aniket Jadhav, Ashwini Gaikwad, Madhura A Jadhav, Mrunal Shinde

**Affiliations:** 1 Department of Conservative Dentistry and Endodontics, Bharati Vidyapeeth deemed to be University Dental College and Hospital, Pune, Maharashtra, India; 2 Department of Orthodontics and Dentofacial Orthopedics, Bharati Vidyapeeth deemed to be University Dental College and hospital, Pune, Maharashtra, India

**Keywords:** Cone beam computed tomography (CBCT), Indian population, prevalence, second mesiobuccalcanal

## Abstract

The success of endodontic treatment relies on the knowledge of root canal anatomy and the ability to access, clean and fill the entire canal system. Therefore, it is of interest to evaluate the prevalence, position and canal arrangement of the MB2 (mesiobuccal 2) canal in 170 maxillary first molars. The MB2 canal was identified in 59.41% of the teeth, with Vertucci Type II arrangement being the most common (69.3%), followed by Type IV (30.7%). The average inter-canal distance between MB1 and MB2 was 1.84 ± 0.41 mm. Spatial analysis showed that the MB2 canal lies in close proximity to MB1, with no significant association between canal configuration and gender (p = 0.583). Thus, we show CBCT's value in detecting complex canal morphology and the need for population-based studies in diverse populations like India to improve endodontic predictability.

## Background:

The success of endodontic treatment relies on the knowledge of root canal anatomy and the ability to access, clean and fill the entire canal system. Maxillary first molars, the most commonly treated teeth, show high endodontic failure rates mainly due to missed canals, particularly the MB2 canal [1, 2]. The mesiobuccal root of maxillary first molars shows complex canal variations and missed or inadequately cleaned canals can compromise treatment outcomes [3]. The variations in the prevalence of the MB2 canal have been found to vary from less than 30% to over 90% in previous anatomical and clinical studies, globally [4, 5]. Moreover, conventional radiography has limited ability to detect complex anatomy due to its two-dimensional nature and image distortion. Hence, these factors often lead to the underestimation of anatomy in teeth with canals that are closely located or fused together [6]. Cone-beam computed tomography (CBCT) is a valuable diagnostic tool in endodontics, providing 3D visualization of root canal anatomy with minimal distortion. This enables accurate assessment of canal prevalence, spatial relationships and configurations of the MB2 canal [7]. Additionally, canal configuration systems such as vertical's classification standardize the description of root canal morphology and enable reliable comparative anatomical analysis [8]. Root canal internal anatomy varies among populations and ethnic groups, highlighting the need for population-specific anatomical data [9]. India is highly heterogeneous, yet data on the prevalence, location and configuration of the MB2 canal across its sub-populations remain limited. Therefore, it is of interest to evaluate these parameters in maxillary first molars of the Western Maharashtra population.

## Materials and Methods:

This retrospective radiographic study utilized CBCT scans from the institutional records of the Department of Oral Medicine and Radiology. A total of 170 CBCT scans were selected after applying predefined inclusion and exclusion criteria. Scans of completely erupted maxillary first permanent molars from males and females aged 15-75 years were included. Scans with incomplete records, poor image quality or artifacts and previously endodontically treated teeth were excluded. All scans were acquired using a CS 9600 Carestream CBCT unit (Carestream Dental, France; 2018). The imaging parameters were 120 kV, 6-8 mA, 10 x 5 cm field of view and 0.12 mm voxel size. Image interpretation was performed using CS Imaging software (version 8.0.20each scan was independently assessed by two examiners to record MB2 canal prevalence, orifice position and morphology in maxillary first molars. MB2 prevalence was assessed using sequential axial slices from the pulp chamber floor to the mesiobuccal root. Findings were corroborated with sagittal and coronal views to confirm canal continuity and merging with MB1. The spatial relationship of the MB2 orifice to MB1 and palatal canals was measured on axial sections. All measurements were recorded in millimetres using CBCT software tools. When present, the MB2 canal course was traced from the pulp chamber to the apex using multi-planar reconstructions. Canal morphology was classified according to Vertucci's classification for standardized assessment. All data were entered into Microsoft Excel (version 2017) and analyzed using SPSS (IBM Corp., version 26.0). Descriptive statistics assessed prevalence, canal configuration and anatomical location. The chi-square test evaluated associations between gender and canal configuration. Statistical significance was set at p < 0.05 with a 95% confidence interval.

## Results:

This study investigated the prevalence, anatomical configuration and spatial relationships of the second mesiobuccal (MB2) canal in maxillary molars within an Indian subpopulation using CBCT. Table 1 (see PDF) indicates, the mean (SD) age of the study participants was 29.37 (8.334) years, with the minimum age of 17 years and a maximum age of 54 years. Table 2 (see PDF) shows the gender distribution, with females comprising 64.4% and males 35.6% of the study sample. The prevalence of the MB2 canal is shown in Table 3 (see PDF), the MB2 canal was found to be present in 101 teeth (59.41%) and absent in 69 teeth (40.59%). The distribution of Vertucci canal types in 101 MB2 canals is shown in Table 4 (see PDF). Type II canal type was found in 70 canals, accounting for 69.3%, whereas Type IV canal type was found in 31 canals, accounting for 30.7%. This suggests that Type II canal type is the most common type of canal in MB2 canals, implying a high incidence of canal merging before exiting at the apex. Table 5 (see PDF) presents the descriptive statistics of MB2 spatial relationships relative to surrounding anatomical landmarks in 101 participants. The mean distance from the MB2 canal to the palatal canal (MB2-PT) was 0.72 mm, ranging from 0.29 mm to 1.52 mm, indicating relatively close proximity with limited variability (SD = 0.25 mm). In contrast, the distance from the MB1 canal to the palatal canal (MB1-PP) exhibited a wider range, with a mean of 6.79 mm (min = 4.79 mm, max = 9.09 mm, SD = 0.81 mm). This reflects greater anatomical variability in this dimension. The inter-canal distance between MB1 and MB2 averaged 1.84 mm, ranging from 1.01 mm to 3.22 mm, with a standard deviation of 0.41 mm. Table 6 (see PDF) shows the distribution of Vertucci's canal types (Type II and Type IV) in comparing male and female subjects. Of the 70 cases with Type II canal type, 25 were male and 45 were female. Of the 31 cases with Type IV canal type, 11 were male and 20 were female. Although Type II canals were more common in both genders, the gender-wise difference was not statistically significant (p = 0.583). This suggests that Vertucci's canal type is not related to gender.

## Discussion:

In our study based on CBCT analysis, the mesiobuccal second (MB2) canal was identified in 101 out of 170 maxillary first molar samples, which revealed a prevalence of 59.41%. This finding is in close agreement with the previous CBCT studies conducted in the Indian subcontinent, which have shown the prevalence of MB2 to range between 56% and 62% [10, 11- 12]. The current prevalence is slightly lower than that reported in East Asian populations, in which the prevalence of MB2 canal has been found to be 75%-80% [12]. But higher than that reported in some Middle Eastern and European populations, in which the prevalence is generally between 40% and 55% [13]. These variations may relate to ethnicity, genetics, age and imaging methods, underscoring the need for population-specific anatomical databases for endodontic care [14, 15]. Analysis of canal configuration showed that Vertucci Type II configuration was the most common, accounting for 69.3% of MB2 canals. Conversely, Vertucci Type IV configuration, which involves two separate canals running independently from the pulp chamber to the apex, was found in 30.7% of the canals. A similar prevalence of Type II canal configuration has been reported in Turkish and East Asian (Korean) populations based on CBCT and clinical studies [16, 17]. Clinically, this configuration complicates apical debridement, as merged canals may retain residual microorganisms if inadequately instrumented and irrigated. Type IV configurations, although less frequent, require meticulous negotiation, shaping and obturation of two separate canals to avoid treatment failure [18]. There was no statistically significant difference between gender and canal configuration (p = 0.583).The mean MB1-MB2 inter-orifice distance was 1.84 mm, confirming that MB2 is typically palatal and slightly mesial to MB1. The MB2-palatal canal distance was smaller than the MB1-palatal distance, supporting a predictable triangular orifice configuration on the pulp chamber floor. These findings align with Su *et al.* who reported MB2 consistently palatal and slightly mesial to MB1, reinforcing the triangular orifice pattern [19]. Görduysus *et al.* recommended troughing along the mesiopalatal groove with palatal access extension to improve MB2 localization and negotiation [20]. The relatively young mean age (29.37 years) may have increased MB2 detectability, as age-related dentin deposition and calcification reduce canal visibility [21]. CBCT overcomes diagnostic limitations of conventional radiography by providing three-dimensional visualization without anatomical overlap [22, 23]. CBCT reliably depicts canal continuity, convergence and orientation, aiding diagnosis in both primary and retreatment cases [24]. This study advances understanding of mesiobuccal root anatomy in maxillary first molars. It confirms the high prevalence and predominant Vertucci Type II configuration of the MB2 canal using CBCT. It provides clinically relevant data on its predictable spatial location, aiding more accurate canal detection and treatment planning. Further large-scale, multicenter studies across diverse populations are needed. This will strengthen population-specific anatomical databases and improve the precision of endodontic diagnosis and outcomes.

## Conclusion:

We show that the mesiobuccal root of the maxillary first molar has complex anatomy that must be carefully considered during endodontic treatment. Awareness of population-based canal prevalence, position and configuration should guide access modification, canal negotiation and improved cleaning and obturation. Integrating this anatomical knowledge into training and practice can reduce missed canals and treatment failures. Correlating canal morphology with clinical outcomes may further improve evidence-based predictability.
